# Editorial: Restricted repetitive behavior in neurodevelopmental disorders

**DOI:** 10.3389/fnbeh.2023.1197316

**Published:** 2023-04-18

**Authors:** Mark H. Lewis, Michael E. Ragozzino, Takahiro Soda

**Affiliations:** ^1^Department of Psychiatry, University of Florida, Gainesville, FL, United States; ^2^Department of Psychology, University of Illinois at Chicago, Chicago, IL, United States

**Keywords:** mouse models, circumscribed interests, autism, Fragile X, behavioral inflexibility, serotonin receptors, deer mice, stereotypy

Restricted, repetitive behavior (RRB) represents a broad class of responding ranging from repetitive sensory-motor acts (e.g., stereotypies) to behaviors reflecting insistence on sameness or resistance to change (e.g., compulsions, rituals) to highly circumscribed interests (Bodfish et al., 2000). In addition to being rigid or inflexible, RRB exhibits little variation in form of expression and identification of its purpose or function has been elusive. RRB is observed in a number of neurodevelopmental disorders (e.g., intellectual disability, obsessive-compulsive and related disorders, tic disorders) and is diagnostic for autism (Whitehouse and Lewis, 2015; Tian et al., 2022). Despite its clinical importance, much is yet to be learned about its genesis or etiology, maintaining factors, and neurobiological mediation. Not surprisingly then, effective biomedical treatments are lacking. There have been few attempts to bring together and integrate findings from studies of RRB that cut across clinical and pre-clinical areas of investigation (Langen et al., 2011a,b). This is a pressing need, and the aim of this Research Topic is to highlight the transdiagnostic features of RRB and examine the translational value of animal models that have a robust repetitive behavioral phenotype. Thus, the specific themes of this collection include the transdiagnostic nature of RRB, integration of clinical and pre-clinical investigations, and the identification of the neurobiological mechanisms that appear to mediate these behaviors. Three of the contributions involve human participant research. The first is focused on RRB in a diverse sample of children that while experiencing behavioral, emotional and/or cognitive difficulties did not carry a diagnosis, nor were they selected for RRB. The second focuses on circumscribed interests in children with autism. The third queries caregivers and self-advocates about behavioral inflexibility in Fragile X syndrome. A fourth contribution reflects a truly translational approach and extends the previous contribution by also focusing on cognitive inflexibility in Fragile X syndrome using methodologies equally applicable to both human and animal participants. The final three contributions involve pre-clinical studies of RRB. These contributions provide important information on the potential role of serotonin receptors in RRB, probe the expression of RRB using behavioral and pharmacological tools, and determine the role of specific cell types in the prefrontal cortex.

Clinical studies of RRB have typically focused on participants with specific diagnoses (e.g., autism) or typically developing children. Keating et al. examine RRB in children with behavioral, emotional and/or cognitive difficulties but with no diagnosis or selection for RRB. Importantly, significant correlations were found between RRB and the strengths and difficulties questionnaire (SDQ) as well as anxiety. This work represents an important extension of the existing literature and highlights a valuable alternative to focusing on diagnostic categories. These findings also highlight the importance of associating RRB with more general measures of emotional and behavioral difficulties.

Circumscribed interests (CI) involve two sub-domains, restricted interests (RI) and unusual interests (UI) (Uljarević et al., 2022). Spackman et al. have extended this work to explore whether there are distinct individual level profiles of CI in individuals with autism and how such variability associates with demographic, cognitive and clinical variables. This work identifies novel associations and represents the first person-centered approach to characterize the heterogeneity in CI.

Behavioral inflexibility is a hallmark of Fragile X syndrome (FXS) (Reisinger et al., 2020). Jones et al. seek to develop a reliable and valid measure of this behavioral domain and determine the level of interference in daily life due to such inflexibility. The authors used focus groups of caregivers and self-advocates to gather information about the experiences of participants with this behavioral domain in order to create a caregiver/self-report measure. Such an instrument will be of important use in monitoring behavioral inflexibility over time, the extent to which caregivers accommodate to such behaviors and to evaluate response to treatment.

In the case of the Schmitt et al. contribution, behavioral inflexibility associated with Fragile X was assessed using an experimental task. Probabilistic reversal learning was employed as a translational paradigm to assess cognitive flexibility in both participants with FXS and in a mouse model of this disorder. Strikingly similar results were found across species. Including the same sex-dependent profile, using directly translatable bidirectional methodologies is a powerful approach to understanding the neurobiology and developing efficacious treatments.

A different autism relevant mouse model (CNTNAP2) was used by Ghandi et al. to assess the role of parvalbumin (PV) interneurons in the prefrontal cortex (PFC) and the perineuronal nets that enwrap them. Alterations in these interneurons have been linked to ASD relevant deficits. The authors discovered that mice with this genetic mutation exhibited an overexpression of PV interneurons and their perineuronal nets. Transient removal of perineuronal nets improved social interaction in male mice but had no effect on RRB. This work highlights the importance of determining mediators of cortical excitatory/inhibitory imbalance.

A wide variety of pharmacological agents have been tested in neurodevelopmental disorders, but as yet, no drug has demonstrated efficacy in attenuating RRB. There is a great need, therefore, to evaluate druggable targets that look promising for clinical application. Alvarez et al. provide an informative review of the effects of modulating serotonin receptor sub-types on repetitive sensory motor behaviors in rodents. As they point out, the picture is far from clear. A number of 5-HT receptor targets have received little attention and should be the focus of future work. It also may be that pharmacological agents that impact multiple neurotransmitter receptors may ultimately prove more effective in treating RRB.

A number of genetic mutant mouse models, including two featured here, display RRB. RRB is also characteristic of animals maintained in restricted environments. Burke et al. employ the repetitive motor behavior typical of deer mice housed in standard lab cages to test different neurocognitive explanations for the expression of RRB (e.g., escape, coping with stress). Both environmental and pharmacological manipulations were employed to this end. This contribution reflects important ongoing efforts in both clinical and pre-clinical studies to determine the motivational basis that might explain robust expression of behaviors that appear to have no obvious function.

## Author contributions

ML drafted the editorial. MR and TS revised the editorial. All authors made a direct contribution to the work and approved it for publication.
